# Descriptive analyses of maternally-derived antibody levels against porcine circovirus 2 (PCV-2) in 3- and 21-day-old piglets from farms of four European countries using different vaccination protocols in sows

**DOI:** 10.1186/s40813-022-00284-9

**Published:** 2022-10-03

**Authors:** M. Sibila, A. Llorens, E. Huerta, C. Fablet, M. Faderl, L. Ferrari, N. Rose, A. Palzer, P. Martelli, M. C. Venegas-Vargas, D. Fredrickson, L. Taylor, M. Balasch, M. Bandrick, J. Segalés

**Affiliations:** 1 Unitat Mixta d’Investigació IRTA-UAB en Sanitat Animal, Centre de Recerca en Sanitat Animal (CReSA), Campus de la Universitat Autònoma de Barcelona (UAB), 08193 Bellaterra, Catalonia Spain; 2 IRTA, Programa de Sanitat Animal, Centre de Recerca en Sanitat Animal (CReSA), Campus de la Universitat Autònoma de Barcelona (UAB), 08193 Bellaterra, Catalonia Spain; 3 OIE Collaborating Centre for the Research and Control of Emerging and Re-Emerging Swine Diseases in Europe (IRTA-CReSA), Bellaterra, Barcelona Spain; 4 ANSES, Laboratoire de Ploufragan-Plouzané-Niort, BP 53, 22440 Ploufragan, France; 5 Veterinary Pig Practice Scheidegg, Bahnhofstrasse 30, 88175 Scheidegg, Germany; 6 Department of Veterinary Science, University of Parma, Strada del Taglio 10, 43126 Parma, Italy; 7 Zoetis Inc., 333 Portage St, Kalamazoo, MI 49007 USA; 8 Zoetis Manufacturing and Research Spain S.L. Ctra, Camprodon s/n, Finca La Riba, 17813 Vall de Bianya, Girona, Spain; 9 Departament de Sanitat I Anatomia Animals, Facultat de Veterinària, Campus de la Universitat Autònoma de Barcelona (UAB), 08193 Bellaterra, Catalonia Spain

**Keywords:** Porcine circovirus 2, Maternally-derived antibodies, Sow vaccination

## Abstract

**Background:**

Up to now, information on the levels of maternally-derived antibodies (MDA) against PCV-2 in suckling piglets born to sows vaccinated with different strategies is scarce in the literature. In the present observational study, the PCV-2-specific MDA titres from piglets from 109 farms (thirty 3-day-old and thirty 21-day-old piglets per farm) across four different European countries (France n = 30, Germany n = 27, Italy n = 22 and Spain n = 30) using different sow vaccination strategies (during gestation, as a gilt, as a piglet or never) were assessed.

**Results:**

In all four countries, mean log PCV-2 MDA titres were higher in 3-day-old piglets than in the 3-week-old ones, being significant in most of all the comparisons performed. Within each country, the highest PCV-2-specific MDA titres were observed in the 3-day-old piglets born to sows vaccinated during gestation. Indeed, in the four countries, more than 60% of this subpopulation (3-day-old piglets from sows vaccinated during pregnancy) had the highest log PCV-2 titres detectable with the ELISA technique used in this study. The lowest MDA titres were more variable. Whereas in France and Germany the lowest titres corresponded to 21-day-old piglets born from sows vaccinated as a piglet, in Italy, they corresponded to 21-day-old piglets derived from sows vaccinated as a gilt and in Spain to 21-day-old piglets born from non-vaccinated sows. In this study, PCV-2-specific MDA titres at 3 and 21 days of age were not affected by sow parity.

**Conclusions:**

Data obtained could be considered as a European global overview of PCV-2-specific MDA titres present in the pre-vaccinated piglet populations in different European countries, with titres tending to be higher in younger piglets, but with values variable among countries and sow vaccination strategies.

**Supplementary Information:**

The online version contains supplementary material available at 10.1186/s40813-022-00284-9.

## Background

Porcine circovirus 2 (PCV-2) is the causal agent of several clinical conditions known as porcine circovirus diseases (PCVDs), which include systemic disease (PCV-2-SD), reproductive disease (PCV-2-RD), porcine dermatitis and nephropathy syndrome (PDNS), and subclinical infection (PCV-2-SI) [1]. Vaccination is the most common and effective tool to control the clinical outcomes of these diseases [2].

PCV-2 vaccination can be applied to sows/gilts, sows and piglets, or piglets. From all these combinations, the most widely used at the field level is the immunization of piglets around weaning, but its application in sows is increasing worldwide [3]. Vaccination of sows against PCV-2 may seek two objectives. First, sow vaccination may protect gestating animals against PCV-2-RD; in this scenario, the vaccine should be applied during acclimatization, prior to mating, during lactation, or at weaning. Sow vaccination can also be applied to transfer humoral and cellular immunity to the offspring; in this situation, the vaccination takes place during late gestation [2]. A double vaccination program (sow/gilt and piglet) is increasingly practiced under field conditions. In these latter cases, the level of PCV-2-specific maternally-derived antibodies (MDA) at the time of piglet vaccination is paramount as it may potentially influence efficacy (mainly in terms of seroconversion) of piglet vaccination [4–12]. The risk of potential MDA interference on vaccine efficacy in terms of average daily weight gain (ADWG) is considered negligible based on published literature [3, 12]. However, it is very likely that non-successful experimental or field trials have not been published in peer-reviewed journals.

The level of MDA the piglets can have at the time of immunization depends mainly on two factors: (1) the sow immunological status, which in turn depends on the sow vaccination status and/or infection pressure at the farm, and (2) the amount of colostrum intake, hardly measurable and controllable on an individual basis [13]. The level of MDA gradually wanes (in the absence of natural infection or vaccination); waning has been reported to be between 4 and 12 weeks of age for PCV-2 but depends on the level of MDA ingested via colostrum [8–11, 14]. The estimated half-life of PCV-2-specific MDA varies from 16 to 45 days depending on the serological test used and the method selected for its calculation [4, 10, 14, 15]. Up to now, there is a lack of information on the levels and decay rate of PCV-2-specific MDA antibodies in piglets born to sows of various PCV-2 vaccination strategies.

The present study aimed to evaluate the PCV-2 MDA titres in 3- and 21-day-old piglets born to sows/gilts from farms located in four European countries, using different vaccination strategies. Moreover, the decay of these PCV-2 MDA titers (as reduction of PCV-2 MDA titres from 3 to 21 days in piglets per farm) was estimated.

## Materials and methods

### Study design

A total of 109 herds from four European countries (France n = 30, Germany n = 27, Italy n = 22 and Spain n = 30) were selected based on the following sow PCV-2 vaccination strategies: (1) during pregnancy (as a sow): sows were vaccinated at least one time during the gestation of their current litter; they may also have been vaccinated as piglets and gilts; (2) during gilt development (as a gilt): animals were vaccinated during gilt development prior to entering the sow herd and prior to breeding; gilts were not further vaccinated as sows but may have been vaccinated as piglets; (3) as piglet only (as a piglet): sows/gilts were vaccinated against PCV-2 as a piglet around the time of weaning or earlier and no sow/gilt vaccination was further performed; and (4) never: sows/gilts had never been vaccinated for PCV-2 during their lifetime. The PCV-2 vaccines used in the tested farms included all those available in Europe at the time of sampling (years 2018 and 2019).

Within each herd, a cross-sectional study was designed, with 30 piglets at 3 ± 3 days of age and 30 at 21 ± 3 days of age sampled during a one-day visit to the farm (n = 60 piglets per farm). Within each sampling age, and when possible, the sampled piglets belonged to two litters of primiparous (n = 6 piglets per sampling age/farm) and to eight litters of multiparous sows (parity 2 or higher, n = 24 piglets per sampling age/farm). Individual piglets were sampled in a manner to limit bias: small, medium, and large pigs were sampled per litter. No data on cross-fostering was recorded in any of the studied farms.

During the visit to the farm, information on management (type of production system, source of breeding gilts, number of sows on site and type of sow housing), sanitary status (history of PCV-2-SD or other disease outbreaks in the past two years and clinical signs associated to these scenarios), and PCV-2 vaccination schedule (PCV-2 vaccine used, number of doses and schedule of doses) were recorded (when possible).

This study protocol (BH25R-XG-16-658) was reviewed and approved (July 2019) by the Zoetis Ethical Review Board. In addition, owner consent from each farm was received before sample taking.

### Samples

Blood samples were collected and transported refrigerated to each country designated laboratory (Anses Ploufragan-Plouzané-Niort laboratory, [France], Veterinary Practice Scheidegg [Germany] and Department of Veterinary Science, Parma, [Italy]) where were allowed to clot and centrifuged. Individual serum samples obtained were stored at – 20 °C until submission to IRTA-CReSA laboratory (Barcelona, Spain).

### PCV-2 ELISA

Individual serum samples were tested in duplicate (in different ELISA plates) using the SERELISA® PCV-2 Ab Mono Blocking diagnostic kit (Synbiotics). In each plate, in addition to the positive and negative controls included in the kit, one positive and one negative internal reference sample was tested. Serum samples were processed in duplicate following the quantitative method (using a single dilution 1:1000) according to the manufacturer’s instructions. The sample-to negative corrected ratio (SNc) was calculated for each well. PCV-2 antibody titres were inferred introducing the duplicates SNc mean into the formula provided by the kit. When the mean SNc_(1:1000)_ was < 0.051, the titre was considered + 2484 (saturation titre). When mean SNc_(1:1000)_ was > 0.7437 the titre was considered ≤ 150. Results were expressed as back-transformed PCV-2 mean titres ± standard deviation (SD).

### Statistical analyses

Data summaries and descriptive analyses were performed with SAS Program (SAS Institute, Cary, NC). For statistical purposes, PCV-2 titres classified as “ + 2484” and “ ≤ 150” were considered as “2484” and “75” and parity data were re-classified in two categories: 1 and > 1. Prior to analyses, PCV-2 titres were transformed to log. Since the farm was considered the experimental unit, means were calculated for each farm within each country, parity class (1, > 1), and treatment.

The mean Log PCV-2 antibody titres were analysed using a general linear mixed model with fixed effects: country, parity (1, > 1), treatment, all 2-way, and the 3-way interaction and random effect: residual. Pair-wise treatment comparisons were made after testing for a significant (*P* ≤ 0.05) 3-way interaction, 2-way interactions, and main effects. Since the highest order interaction that was significant was the country by treatment, the difference between log least square mean of PCV-2 antibody titres in piglets of 3 and 21 days of age (piglet ages at sampling) within each farm, sow vaccination strategy, and country was calculated. The difference (decay) in PCV-2 antibody titres at piglet age of blood collection were compared within each country with estimate statements. The 95% confidence intervals of the least squares mean Log PCV-2 antibody titre within each country, vaccination strategy and sampling age were calculated.

## Results

### Farm characteristics

The number and type of farms per treatment type and country included in the study are detailed in Table 1. Specific information on management practices, sanitary status and PCV-2 vaccination schedules of the farms is included in Additional files 1, 2, 3, 4, 5: Tables S1–S5.

**Table 1:** Number of samples per country, vaccination strategy, parity, and age groups

Country	Vaccination treatment	3 days of age			21 days of age		
		P 1	P > 1	Total	P 1	P > 1	Total
France	As a sow (n = 9)	54	216	270	54	216	270
	As a gilt (n = 9)	54	216	270	54	216	270
	As a piglet (n = 9)	54	216	270	54	216	270
	Never (n = 3)	18	72	90	18	72	90
Germany	As a sow (n = 8)	36	204	240	48	192	240
	As a gilt (n = 8)	33	207	240	36	204	240
	As a piglet (n = 9)	57	213	270	39	231	270
	Never (n = 2)	15	45	60	18	42	60
Italy	As a sow (n = 3)	18	72	90	18	72	90
	As a gilt (n = 8)	42	168	210	42	168	210
	As a piglet (n = 8)	57	213	270	54	216	270
	Never (n = 3)	18	69	87	18	68	86
Spain	As a sow (n = 9)	54	216	270	54	216	270
	As a gilt (n = 9)	54	216	270	54	216	270
	As a piglet (n = 9)	54	216	270	54	216	270
	Never (n = 3)	18	72	90	18	72	90

*P* parity.

### Distribution of PCV-2 MDA titres among the tested population

**Fig. 1:**
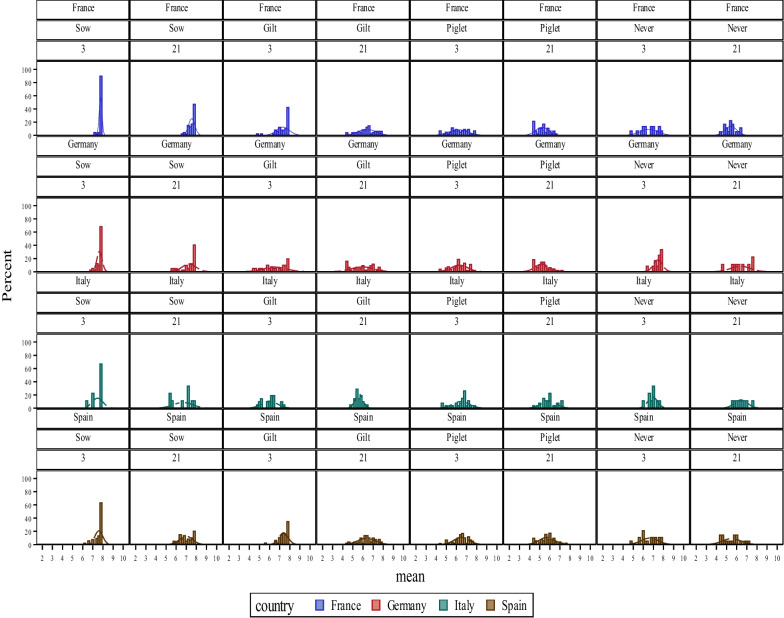
Distribution (in percentages) of log least-square mean of PCV-2 titres in 3- and 21-day-old piglets per vaccination strategy and country

Distribution of log least square mean PCV-2 specific titres of 3- and 21-day-old piglets per treatment and country are represented in Fig. 1. In the four countries, more than 60% of the 3-day-old piglets born to sows vaccinated during pregnancy had the highest log PCV-2 titres detectable with the ELISA technique used in the present study (8 log). Such predominance (but much less pronounced) was also observed in France and Germany in 21-day-old piglets born from sows vaccinated during gestation and in Spain and France in 3-day-old piglets born from sows vaccinated as Gilts. On the contrary, in Italy, the predominance of piglets with very high PCV-2 titres was not detected in any other treatment and age-group.

### Mean PCV-2 MDA titres per age, vaccination strategy, and country

In all four countries, mean log PCV-2 specific MDA titres were higher in 3-day-old piglets than in the 3-week-old ones, being statistically significant in all the comparisons performed but in Germany in those sows vaccinated as a Gilt and in Italy in those vaccinated as a Gilt or Never vaccinated (Additional file 6: Fig. S1).

**Fig. 2:**
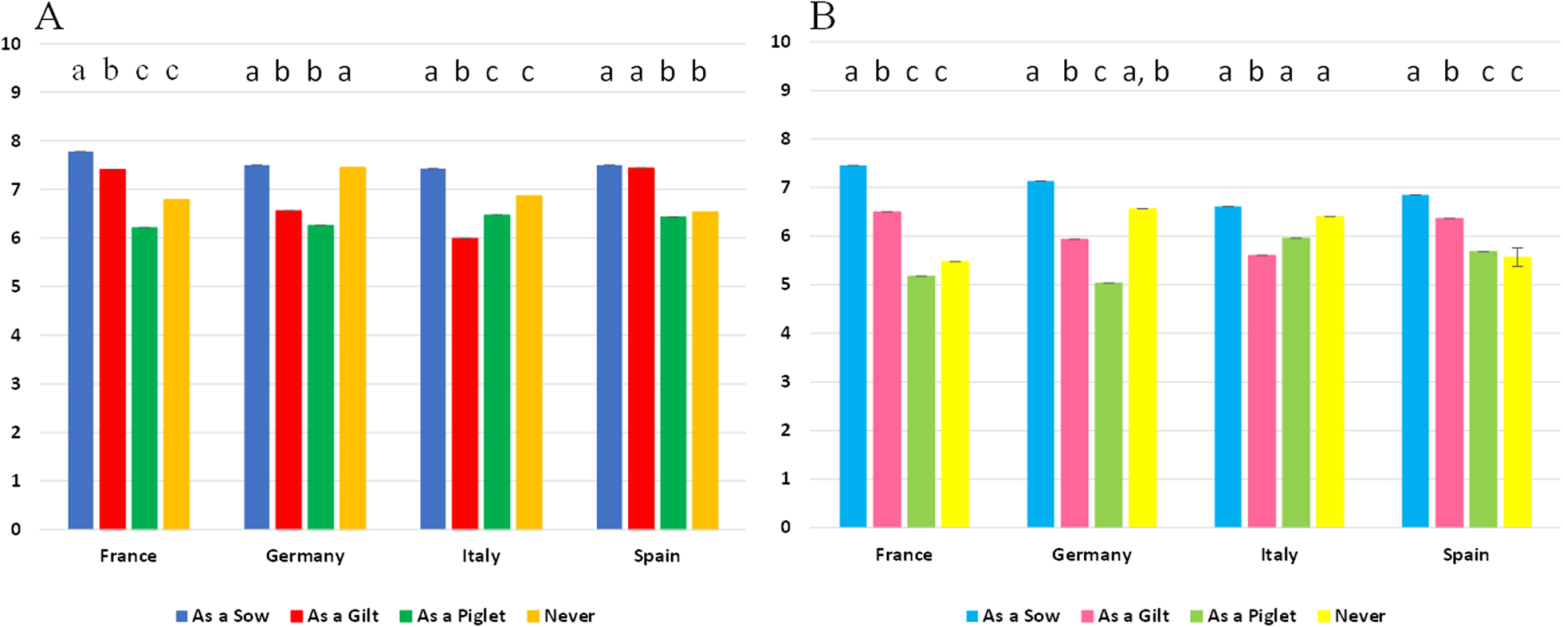
Log least square mean [± SD] of PCV-2 titres at 3 (**A**)- and 21 (**B**)-day-old piglets per vaccination regimen of the sow and country. Different letters mean *p* < 0.05 between treatments within a country

Within 3-day-old piglets (Fig. 2A), piglets born to sows vaccinated during gestation showed higher PCV-2 specific MDA titers when compared to the other vaccination strategies but with the following exceptions: in Germany when compared to piglets born from sows never vaccinated and in Spain when compared to piglets born from sows vaccinated as a gilts. At this age, the numerically lower PCV-2 MDA titers were observed in piglets born from sows vaccinated as a piglet (in France, Germany, and Spain) or as a gilt (Italy).

In 3-week-old piglets, the situation was like the one depicted above but reached lower PCV-2 titers (Fig. 2B). The numerically higher PCV-2 MDA titers were also observed in piglets born from sows vaccinated during pregnancy in all countries and the numerically lower ones in piglets derived from sows vaccinated as a Piglet (France and Germany), as a Gilt (in Italy) and in sows never vaccinated or vaccinated as a Piglet (Spain).

### PCV-2 MDA decay from 3 to 21 days of age per farm

**Fig. 3:**
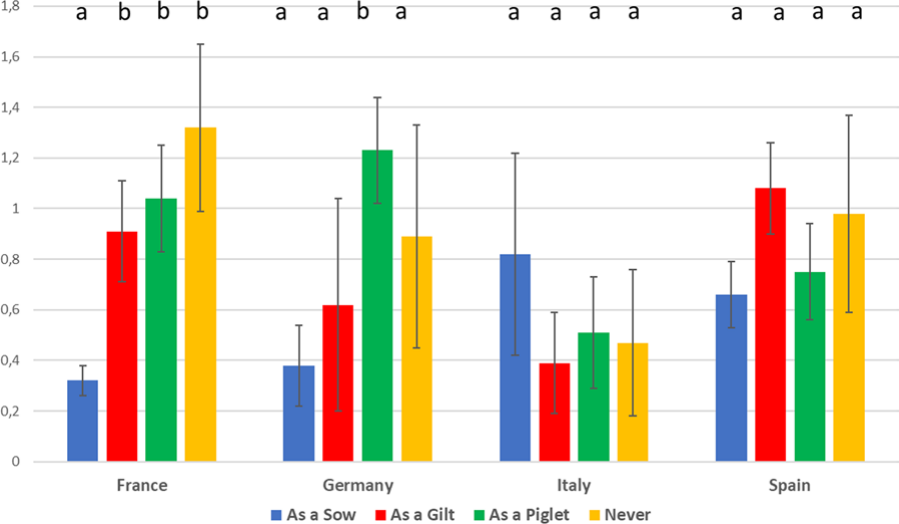
MDA decay (as difference of Log least square mean [± SD] PCV-2 titres from 3 to 21 days of age) per vaccination regime of the sow within each country. Different letters mean p < 0.05 among different vaccination regimen in a country

In France, the reduction of PCV-2 MDA titres between piglets of 3 and 21 days-of-age was significantly lower in piglets coming from sows vaccinated during pregnancy compared to the other sow vaccination regimes (Fig. 3). In Germany, the reduction in MDA showed a similar profile, but the significant differences were only observed between piglets from sows vaccinated during pregnancy and from those born to sows vaccinated only as a piglet. Although in Italy and Spain no statistically significant differences in PCV-2 MDA decay between vaccination regimes were detected, a different pattern was observed. Whereas in Italy the highest reduction was observed in piglets derived from sows vaccinated during pregnancy, in Spain the highest reduction was observed in piglets derived from sows vaccinated as a gilt.

### PCV-2 MDA titres versus parity and sow vaccination strategy (per country)

No statistically significant differences in PCV-2-specific MDA levels between 3-day-of-age piglets from sows of different parity (= 1 and > 1) across each vaccination regimen or country were found.

## Discussion

The present observational study provided descriptive data on PCV-2 specific MDA titres from 109 farms from four different countries using different sow vaccination strategies. The number of farms tested in each country ranged from 22 to 30. Thirty animals of 3 and 30 of 21 days of age born from sows of different parity numbers were tested from each farm. Overall, the data obtained could be considered as an overview of PCV-2-specific MDA titres present in the pre-vaccinated piglet populations in different European countries.

Up to now, the prevalence of PCV-2 infection in young piglets (< 3 weeks of age) has been assessed in different studies with the goal of describing pre-vaccination infection rates [16, 17]. However, information on PCV-2-specific MDA titres during the lactation period is very limited [7]. This information might be of interest to assess the level of MDA transferred to the offspring through colostrum intake under different PCV-2 sow vaccination strategies. Indeed, most of the studies available in the literature reporting PCV-2 MDA levels in vaccinated populations were focused on comparing the levels of antibodies present at the time of piglet vaccine administration, mostly around weaning [5–9, 12, 18]. The main objective behind such studies was to assess the effect exerted by certain PCV-2 MDA levels on the efficacy of the administered vaccine (reviewed by Poulsen-Nautrup et al. [3]). As far as the authors’ knowledge, this is the first description of PCV-2-specific MDA titres in 3-day-old and 3-week-old piglets derived from sows vaccinated using different strategies. The fact that PCV-2 titres were measured at 3 and 21 days of age in different animals (cross-sectional study) is a limitation of this work since the individual half-life and MDA decay rate could not be calculated. Nevertheless, the information provided on the PCV-2 MDA reduction from 3 to 21 days of age at the farm level might be useful when planning a sow and/or piglet vaccination strategy.

As expected, vaccinating sows during gestation resulted in a high proportion (> 60%) of 3-day-old piglets having very high antibody titres. Indeed, in these farms, the level of MDA observed in 21-day-of-old piglets was still high (means from 6.65 to 7.82 Log PCV-2 titres, which would be equivalent to 10.32 to > 14.32 Log_2_ immunoperoxidase monolayer assay (IPMA) titres based on the comparison performed by Pileri et al. [19]). PCV-2-specific MDA levels in 21-day-old piglets from sows vaccinated during gestation were higher than those observed in 3-day-old piglets coming from sows vaccinated as a gilt (in three out of four countries). Indeed, PCV-2-specific MDA titres of piglets from vaccinated sows had the lowest titre reduction from 3 to 21 days of age (in all countries but Italy). These results could be explained by two main reasons: (1) a slower waning of MDA when the titres are high; but this rationale should be confirmed by analysing the MDA decay in the same animals (in the present study, the sampled piglets at 3 and 21 days of age were different), and (2) these animals had the highest titre calculated by the ELISA technique used (2484, equivalent to 7.8 Log PCV-2 titres) and, probably, the specific level of antibodies in animals with titres > 2484 could not be determined unless dilutions would have been applied.

In general terms, high titres of MDA (equivalent to ≥ 8.32 Log_2_ IPMA titres) at the time of vaccination have been usually associated to a reduction in vaccine seroconversion [4–6, 8, 9, 12]. Interference with vaccine efficacy, however, was not observed in most of the studies using the ADWG as the primary outcome [5, 7, 12, 18]. Noteworthy, ADWG was affected by MDA when the vaccinated animals had high antibody titres at 1 week of age (> 10.6 – 12.2 Log_2_ ELISA) [7] or when antibody titres were, at 3 weeks of age, extremely high (> 17.32 Log_2_ IPMA titres) [12]. In this latter study, such very high titres were mainly achieved by vaccinating the sows at 3- and 6-weeks pre-farrowing; however, under common commercial conditions it would not be expectable having a high proportion of animals harbouring extremely high titres. In line, the results reported in the present study suggest the number of animals with very high PCV-2 titers elevated enough to interfere with vaccine efficacy in terms of AWDG would not be negligible when the sows are vaccinated. Obviously, this situation will depend on the gestational time of vaccination, since it would not be the same to vaccinate early during gestation or during late gestation [20] and, probably, on the vaccine used. In the present study, information on the exact moment of gestational time of vaccination was not available as sow PCV-2 vaccination was mostly done in a blanket fashion in Italy and Spain; in the case of France sow vaccination was mainly applied before farrowing (range of 2–12 weeks pre farrowing) while in Germany the time of sow vaccination was variable depending on the farm. Therefore, we cannot reach any conclusion on the effect of gestational vaccination timing regarding piglet antibody levels. Further studies comparing the level of MDA in piglets coming from sows vaccinated at early or late gestation would be required. Nevertheless, in a blanket fashion vaccination, all possibilities may be represented [20]; in any case, sow vaccination close to the farrowing time would result in the highest MDA titres in piglets.

Although one might expect that the lowest PCV-2 MDA titres would be found in the 21-day-old piglets from non-vaccinated farms, this only happened in Spain. In France and Germany, the lowest values were detected in 21-day-old piglets from sows vaccinated as piglets, and in Italy they were observed in the 21-day-old piglets born from vaccinated gilts. These results probably reflect the variation in terms of sow PCV-2 natural infection that may have occurred in just a proportion of these farms. Therefore, no sound predictions can be made regarding the piglet PCV-2 specific antibody values at the individual farm level based on the sow vaccination strategy.

This study provides information on the PCV-2 MDA titres in farms where the sows had never been vaccinated. This information might be very valuable as there are very few farms not vaccinating against PCV-2 nowadays [2]. This scenario is reflected by the low number of farms included in this category (France n = 3; Germany n = 2, Italy n = 3 and Spain n = 3), since they were extremely difficult to be found. High MDA levels (especially in 3-day-old piglets) detected in the herds in which the sows had never been vaccinated may indicate a high viral circulation [21, 22].

Although in previous studies sow parity has been linked to different general immunoglobulin concentrations [23], PCV-2 MDA titres in the present study were not different between piglets from primiparous or multiparous sows. It cannot be ruled out that these results have been influenced by the different sample size between multiparous (2633 and 2631 observations at 3 and 21 days of age, respectively) and primiparous (636 and 633 observations at 3 and 21 days of age, respectively) or by the different vaccines used in farms. More studies would be needed to ascertain if PCV-2-specific MDA titres are influenced by the parity, but it is probably more dependent on the particular degree of PCV-2 circulation in the farm rather than sow parity.

In summary, the levels of PCV-2-specific MDA in 3- and 21-day-old piglets varied depending on the sow vaccination strategy applied, although certain differences between sow vaccination regimes were observed among the studied countries. Vaccination of sows during gestation implied a high proportion of piglets with high MDA titres both at 3 and 21 days of age. The impact of these high titres to piglet vaccination efficacy should be further assessed.

## Supplementary Information

**Additional file 1. Table S1.** Characteristics of the 30 French Farms included in this study.

**Additional file 2. Table S2.** Characteristics of the 27 German Farms included in this study.

**Additional file 3. Table S3.** Characteristics of the 22 Italian Farms included in this study.

**Additional file 4. Table S4.** Characteristics of the 30 Spanish Farms included in this study.

**Additional file 5. Table S5.** Number of farms receiving each particular vaccine and doses (in parenthesis) in each category.

**Additional file 6. Figure S1.** Log least square mean of PCV-2 titres in 3- and 21-day-old (d) piglets per vaccination regimen and country. * means p ≤ 0.05 between animals of 3 and 21 days of age within each vaccination regime.

## Data Availability

The datasets used and analysed during the trial are available from the corresponding author on request.

## Abbreviations

MDA: Maternally-derived antibodies
SNc: Sample to negative corrected ratio
