# Supplementary material for: Descriptive analyses of maternally-derived antibody levels against porcine circovirus 2 (PCV-2) in 3- and 21-day-old piglets from farms of four European countries using different vaccination protocols in sows
Source: Porcine Health Manag. 2022 Oct 3;8:41. doi: 10.1186/s40813-022-00284-9 (PMC9531413; doi:10.1186/s40813-022-00284-9)
Supplement: Supplementary file 6 — Additional file 6. Figure S1. Log least square mean of PCV-2 titres in 3- and 21-day-old (d) piglets per vaccination regimen and country. * means p ≤ 0.05 between animals of 3 and 21 days of age within each vaccination regime. [file 40813_2022_284_MOESM6_ESM.docx]

**Additional file 6**

**Figure S1**: Log least square mean of PCV-2 titres in 3- and 21-day-old (d) piglets per vaccination regimen and country. *means p≤0.05 between animals of 3 and 21 days of age within each vaccination regime.


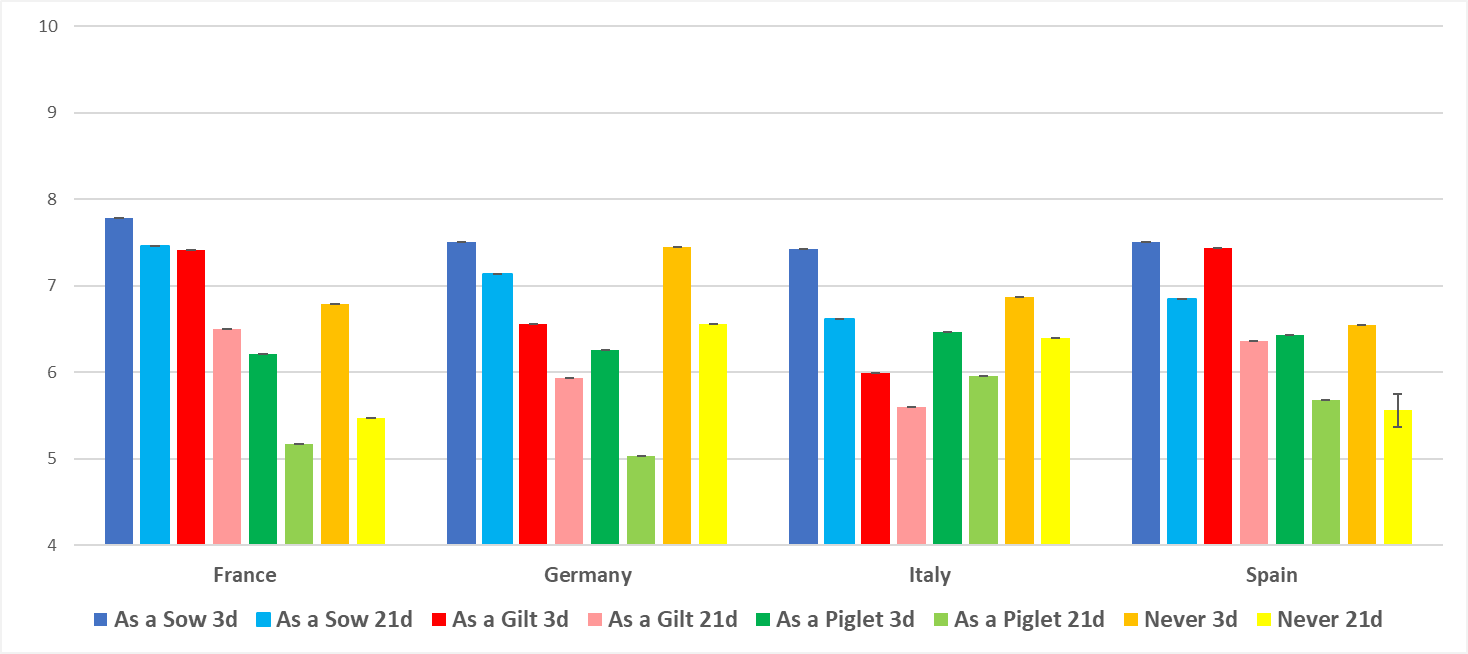


*

*

*

*

*

*

*

*

*

*

*

*

*
